# *Facklamia hominis* pyelonephritis in a pediatric patient: first case report and review of the literature

**DOI:** 10.1186/s12941-022-00497-4

**Published:** 2022-02-12

**Authors:** Samantha Pérez-Cavazos, Daniela Cisneros-Saldaña, Fernando Espinosa-Villaseñor, José Iván Castillo-Bejarano, Denisse Natalie Vaquera-Aparicio, Hugo Sánchez-Alanís, Abiel Mascareñas-De los Santos

**Affiliations:** 1 Department of Pediatrics / Infectious Diseases Service, Hospital Universitario “Dr. José Eleuterio González” Universidad Autónoma de Nuevo León, Francisco I. Madero Avenue, Mitras Centro, ZC 64460 Monterrey, Mexico; 2 Department of Clinical Pathology, Hospital Universitario “Dr. José Eleuterio González” Universidad Autónoma de Nuevo León, Francisco I. Madero Avenue, Mitras Centro, ZC 64460 Monterrey, Mexico

**Keywords:** Facklamia hominis, Urinary tract infection, Emerging pathogen, Child, Children, Pediatric

## Abstract

**Background:**

Pyelonephritis is one of the most serious bacterial illnesses during childhood. Gram-negative organisms account for up to 90% of the cases. Gram-positive bacteria are uncommon causes of urinary tract infections, and only a few cases caused by *Facklamia hominis* have been reported in the literature.

**Case presentation:**

A five-year-old girl with tracheostomy and gastrostomy and past medical history of congenital lymphangioma presented with a two-week history of with intermittent fever, frequent urination, and vesical tenesmus. Diagnosis of pyelonephritis was made. Urine culture reported colonies with alpha-hemolysis in blood agar at 48-h of incubation and *Facklamia hominis* was identified by MALDI-TOF. The patient was successfully treated with gentamicin.

**Conclusions:**

This is the first reported case of pyelonephritis by *Facklamia* hominis in a child, and the second involving infection in a pediatric patient. Although this pathogen is uncommon, current treatment of *F. hominis* is a challenge for physicians. This case illustrates the requirement to standardize identification and treatment of care to avoid treatment failure and antimicrobial resistance.

## Background

Acute pyelonephritis is an infection that affects renal parenchyma and in children, it represents one of the most common causes of serious bacterial illnesses. 80–90% of cases are caused by *Escherichia coli*, and the remaining 10–20% by other Gram-negative bacilli, such as *Klebsiella, Enterobacter, Proteus,* and *Pseudomonas* species, and certain Gram-positive organisms such as *Enterococcus faecalis, Staphylococcus saprophyticus*, and *Streptococcus agalactiae *[1, 2].

*Facklamia* species are rarely involved in human infections. These Gram-negative cocci are alfa-hemolytic, catalase negative, and facultatively anaerobic [3–7]. From six members of this genus, only four (*F. hominis, F. sourekii, F. ignava,* and *F. languida*) have been identified in human clinical specimens, causing a range of illnesses such as sepsis, bacteremia, abscesses, peritonitis, meningitis, endocarditis, genitourinary infection, chorioamnionitis, and prosthetic joint infection [3–6]. *Facklamia hominis* is the most common member of this genus involved in human infection [3]. We present the first reported case of pyelonephritis caused by *Facklamia hominis* in a child.

## Case presentation

**Fig. 1:**
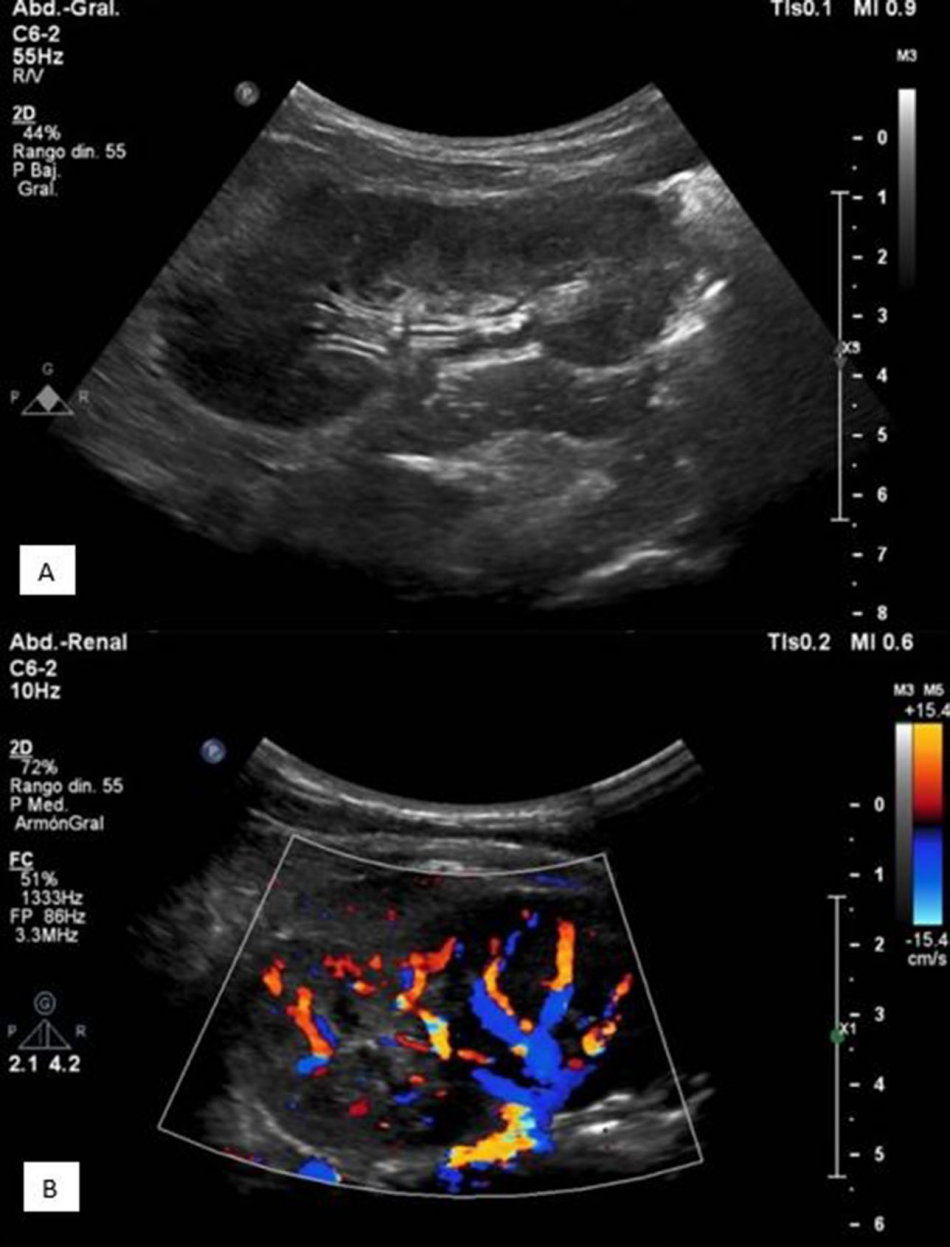
Renal ultrasonography images: showing evidence of focal left pyelonephritis. **A** Echogenic bands in superior pole of left kidney with compression of the renal sinus. **B** Diminished perfusion in the superior pole of left kidney in color-Doppler ultrasound

**Fig. 2:**
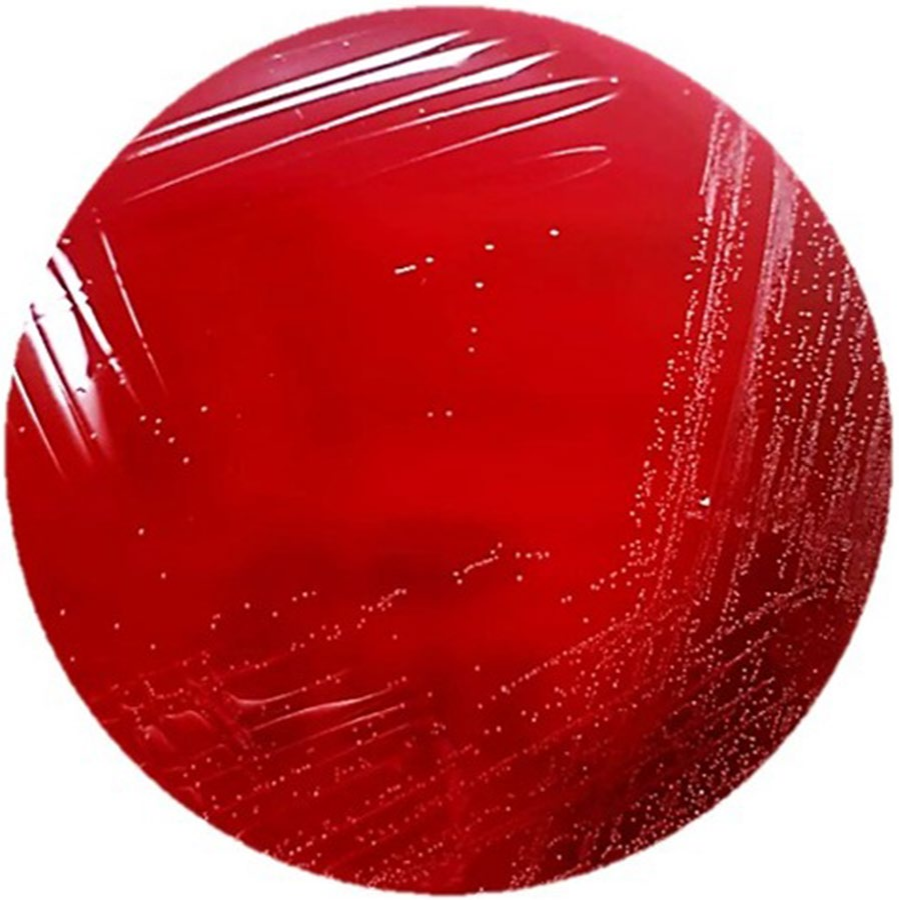
Urine culture: Alpha-hemolytic, non-pigmented colonies in 5% blood sheep agar, after a 48-h incubation period

We present the case of a 5-year-old girl from Mexico, with medical history of a congenital cervical lymphangioma, with a partial surgical resection during the neonatal period, requiring a gastrostomy and tracheostomy, and currently being treated with oral sirolimus (0.8 mg/m^2^/twice a day). She presented to the emergency department with a two-week history of intermittent fever, increased urination frequency, and vesical tenesmus, for which she was treated in an out-patient clinic with levofloxacin for seven days with no clinical response. Upon physical examination she had mild abdominal tenderness and left costovertebral angle tenderness. Complete blood count (CBC) was normal (leukocytes 6.25 k/µL, neutrophils 3.1 k/µL, lymphocytes 2.3 k/µL, hemoglobin 12.6 g/dL, and platelets 249,000/mm3). Acute phase reactants were slightly elevated (C-reactive protein 1.1 mg/dL, erythrocyte sedimentation rate 30 mm/h). Urinalysis was normal (negative nitrites, leukocyte esterase, and no leukocytes). Renal ultrasound showed a normal right kidney, and echogenic bands in the superior pole of the left kidney, in addition to renal sinus compression, and areas of cortical hypoperfusion in color-Doppler examination, suggestive of focal left pyelonephritis (Fig. 1). Blood and urine cultures were taken upon admission, the latter obtained via vesical catheterization. Given that the patient had no history of recent hospitalization, intravenous gentamicin (6 mg/kg/day) was empirically initiated as a second line of treatment for community-acquired pyelonephritis. Blood cultures were negative. After a 48-h incubation period, urine culture reported growth of colonies with alpha-hemolysis on blood agar (> 100,000 CFU/mL) (Fig. 2). Colonies were identified as *Facklamia hominis* by a MALDI-TOF Biotyper® using a matrix-assisted laser desorption/ionization and time-of-flight (MALDI-TOF) mass spectrometry system, with a score value of 2.03. Antimicrobial susceptibility was determined via disc method, and the four antibiotics tested (vancomycin, gentamicin, tetracycline, linezolid) were susceptible. Quinolones were not available in laboratory. The patient presented clinical resolution of symptoms within 72 h of therapy. Due to the rarity of the case, a follow-up urine culture was performed 14-day later, with no growth reported, and she was discharged after completing a two week-course of gentamicin.

## Discussion and conclusions

The genus *Facklamia* was first described in 1997 by Collins et al., using comparative 16S recombinant ribonucleic acid (rRNA) gene sequencing studies, and it currently comprises six species: *F. ignava*, *F. sourekii*, *F. tabaciasalis, F. languida, F. miroungii and F. hominis* [4, 6, 8]. Only *F. tabaciasalis and F. miroungii* have not been isolated from human specimens [3, 8]. Recently, a novel species, *Facklamia lactis* sp. nov. was isolated from a German bulk tank milk [9].

The natural habitat of *Facklamia* species remains unknown, however strains associated to human infection seem to be rare members of commensal microbiota of skin, and the female genitourinary tract [8]. *Facklamia* species are uncommon human pathogens, and since the description of the genus, very few cases of *Facklamia* spp human infections have been reported in medical literature [3, 7, 10]. The pathogenic potential and virulence of this genus is yet to be dilucidated [7].

*F. hominis* has been isolated from several clinical specimens such as urine, vagina, blood, abscesses, synovial fluid, mitral valves, placentas, gastric aspirates, cerebrospinal fluid, and preputial swabs [6–8, 10–14].

In this case, our patient presented with pyelonephritis and inadequate clinical response to initial treatment with quinolones. Normal findings in CBC and the moderate elevation in acute phase reactants could be explained either by the immunosuppressive properties of sirolimus, or due to a partial response to levofloxacin. However, therapy failure cannot be adjudicated to resistance to levofloxacin, as its susceptibility was not tested in the urine isolate due to routine laboratory protocols. In this case it could not be determined if *F. hominis* was part of the vaginal microbiota of the child, as vaginal cultures were not performed upon admission or after treatment, nonetheless follow-up urine cultures were negative.

The role of sirolimus or other immunosuppressive agents in the pathogenicity of *Facklamia* infections is unknown. Sirolimus has been associated with a higher rate of mucosal herpes simplex virus infections, however that association has not been observed with other pathogens such as cytomegalovirus and hepatitis C; also it has been documented to have antifungal-activity in vitro, as it inhibits the growth of some species such as *Cryptococcus neoformans, Candida albicans* and *Aspergillus fumigatus* [15]*.*

We performed a literature search in four languages (spanish, english, german and korean) of core databases including MEDLINE (National Library of Medicine, Bethesda, MD), SciELO (Scientific Electronic Library Online), Google Scholar (Palo Alto, California) and Web of Science (Clarivate Analytics, Philadelphia, Pennsylvania), between 1990 and 2021 using the keywords: “*Facklamia* spp”, “*Facklamia* species” “*F. hominis*”, “*F. languida”,* “*F. ignava”*, “*F. sourekii”*, “*F. miroungii”* and “*F. tabaciasalis”* to identify case reports and case series. References of the selected publications were reviewed to recognize duplicate reports on case series. Twelve cases of infections due to *F. hominis* were identified. A summary of demographic and clinical data, including risk factors and treatment is available in Table 1.

**Table 1:** Summary of *Facklamia hominis* infection risk factors, diagnostic methods, and treatment

Pub year/Place	Gender/Age	Underlying condition	Infection	Sample	Identification Method	Provided antibiotic treatment
2004, UK [10]	F/ 34 Y	Pregnancy	Chorioamnionitis and puerperal bacteremia	Placental tissue swabs and placental tissue	ND	Amoxicillin and clavulanic acid Metronidazole
2010, UK [11]	ND/ ND	Ischemic stroke	Endocarditis	Blood culture	PCR	Gentamicin and Vancomycin
2012, India [12]	M/ 35 Y	Rheumatic heart disease	Endocarditis	Blood culture	Vitek®2 system	Ceftriaxone Gentamicin
2014, Spain [5]	F/ 81 Y	Obesity	Prosthetic joint infection	Periprosthetic femoral Prosthetic tissue, femoral interface membrane, acetabular interface membrane	Vitek®2 system	Amoxicillin Ceftriaxone
2015, France [16]	F/40 Y	None	Scapular abscess	Abscess culture	MALDI-TOF	Pristinamycine
2015, USA [13]	F/41 Y	Recurrent sinusitis	Meningitis	CSF culture	Vitek®2 system	Ceftriaxone
2017, Germany [17]	M/63 Y	Melanoma	Thoracic abscess	Abscess culture	PCR	Clindamycin
2019, Spain [18]	M/9 Y	Phimosis + balanopreputial adhesions	Balanoposthitis	Urethral exudate culture	MALDI-TOF	Amoxicillin and clavulanic acid
2019, Korea [19]	M/67 Y	Hepatitis B virus-cirrhosis	Epidermal cyst	Wound secretion culture	Vitek®2 system	Amoxicillin and clavulanic acid
2020, Switzerland [14]	M/75 Y	Benign prostatic hyperplasia + transurethral prostate resection	Urosepsis	Urine culture	RAST (vClassic RAST)	Ampicillin-Sulbactam Vancomycin
2020, France [6]	F/ 57 Y	Uterine myomatosis and obesity	Peritonitis	Peritoneal culture	MALDI-TOF Vitek MS®	Cefotaxime Metronidazole Amoxicillin
2021, USA (20)	F/64 Y	Type 2 diabetes mellitus and coronary artery disease	Hidradenitis suppurativa	Axillary abscess culture	MALDI-TOF	Moxifloxacin Vancomycin Trimethoprim/Sulfamethoxazole
2021, Mexico	F/ 7 Y	Congenital lymphangioma	Pyelonephritis	Urine culture	MALDI-TOF	Gentamicin

*M* male, *F* female, *CFS* cerebrospinal fluid, *PCR* polymerase chain reaction, *MALDI-TOF* matrix-assisted laser desorption/ionization time-of-flight, *ND* no data

*Facklamia* species are Gram-positive, catalase-negative, facultative anaerobic cocci, and its identification with traditional microbiologic tests can represent a challenge. Depending on species and growth conditions, they can be found in pairs, clusters, or chains, and on sheep blood agar colonies are non-pigmented and weakly alpha-hemolytic or non-hemolytic. Due to its resemblance in Gram-stain characteristics, colony morphology, hemolysis on 5% sheep blood agar, and catalase reaction, they may be misidentified as viridans-group Streptococci or discarded as contaminants [3, 7, 8, 10]. *Facklamia* species can be recognized by their production of leucine aminopeptidase (LAP), L-pyrrolidonyl-beta-naphthylamide (PYR), and their ability to grow in 6.5% sodium chloride containing media [3–7].

Species of *Facklamia* have been identified using the API Rapid ID32 STREP system, API ZYM method, Vitek® 2 system (bioMerieux France), matrix-assisted laser desorption/ionization time-of-flight mass spectrometry (MALDI-TOF), and DNA sequence coding for 16S RNA [6, 8, 14] In the present case, *Facklamia hominis* was successfully identified by MALDI-TOF.

The distinction of *Facklamia* spp from viridans-group Streptococci and other *Streptococcus*-like organisms is clinically relevant because of its unusual susceptibility pattern. Susceptibility testing has been defined using the Clinical and Laboratory Standards Institute Guidelines for *Streptococcus* species other than *Streptococcus pneumoniae*. Physicians must be aware of the several limitations concerning *Facklamia* infections, as to date there is no standardized method of identification, or treatment guidelines available, and taking into account the inconsistency of susceptibility profiles reported, clinical decisions should be taken considering the susceptibility pattern of the isolate in question [7, 8].

Outcomes of *F. hominis* infections are mostly encouraging when appropriate antibiotic therapy is warranted, such as the case we present [6].

## Conclusion

This case is the second reported in a pediatric patient, the first reported in Latin America, and the first case of *Facklamia hominis* pyelonephritis in a child. Although this pathogen is an uncommon cause of infection, its identification and treatment remain a challenge for physicians. A variety of antibiotics have been used, including penicillin and third generation cephalosporins. In our case, the patient was successfully treated with gentamicin. Overall, our attestations point up that *F. hominis* is responsible of a variety of human infections and should be considered as an emerging pathogen, however there is scarce information of its virulence and pathogenic potential. This case illustrates the importance of appropriately identifying the bacteria and its susceptibility patterns, to prevent treatment failure and to avoid selective pressure on microbiota, thus preventing antimicrobial resistance.

## Data Availability

Not applicable.

## Abbreviations

API: Analytical Profile Index
CBC: Complete blood count
CFU: Colony-forming unit
DNA: Deoxyribonucleic acid
LAP: Leucine aminopeptidase
MALDI-TOF: Matrix-assisted laser desorption/ionization time-of-flight
PYR: L-pyrrolidonyl-beta-naphthylamide
RNA: Ribonucleic acid
